# Berry e-liquid flavour toxicities are not equal on an alveolar-capillary barrier cell model according to two exposure methods

**DOI:** 10.3389/ftox.2026.1769275

**Published:** 2026-03-11

**Authors:** Emma Filaudeau, Amelia-Naomi Sabo, Anita Lebert, Laurent Monassier, Véronique Kemmel

**Affiliations:** 1 Laboratoire de Pharmacologie et Toxicologie Neurocardiovasculaire, UR 7296, Centre de Recherche en Biomédecine de Strasbourg (CRBS), Faculté de Médecine, Maïeutique et Sciences de la Santé, Strasbourg, France; 2 Gaïatrend, Rohrbach-lès-Bitche, France; 3 Laboratoire de Biochimie et Biologie Moléculaire, Hôpitaux Universitaires de Strasbourg, Strasbourg, France

**Keywords:** alveolar-capillary barrier, barrier integrity, berry flavours, e-cigarette, oxidative stress, trapped aerosol

## Abstract

E-cigarettes are often presented as a smoking cessation tool, less harmful than tobacco and are now a regular feature of everyday life. However, the fruity and sweet flavours are attractive to teenagers who can use them recreationnaly. The main risk of this phenomenon is becoming trapped in nicotine addiction. Although e-liquids, which combine nicotine, humectants and flavouring agents, seem to affect the pulmonary system and in particular the alveolar-capillary barrier (ACB), data on nicotine-free e-liquids remain scarce. The aim of this work is to study the toxicity of nicotine-free e-liquids/aerosols flavoured with berries (Strawberry, Raspberry, Blackberry, Blueberry) using two exposure methods. First, we validated an *in vitro* model of the ACB using epithelial NCI-H441 and endothelial EA.hy926 cells. Secondly, barrier integrity, production of oxidative species and cytotoxicity were assessed by transepithelial resistance (TER) measurement, MitoSOX® and LDH test, respectively, after exposure to two different methods: dilution of the e-liquid and aerosol trapping in the culture medium. Finally, a non-exhaustive analysis of the chemical compounds found in berry e-liquids was performed to identify potentially toxic compounds specific to certain flavours. The model mimicked phenotypically and functionally the ACB. The two exposure methods tested revealed significant differences in terms of e-liquid/aerosol toxicity on the ACB, probably due to variations in aromatic and degradation products. Flavours are not all equal in terms of cell toxicities, making it essential to chemically identify the compounds responsible for these different degrees of toxicity.

## Graphical Abstract

**Figure:**
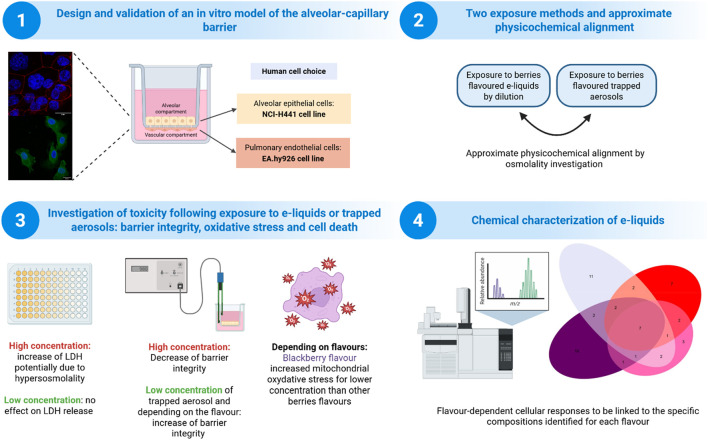

## 1 Introduction

Electronic cigarettes (e-cig) emerged on the market in the 2010s and have often been presented as a less harmful alternative to conventional cigarettes. Initially targeting smokers seeking to reduce or even stop their tobacco consumption, their use of e-cig has gradually been directed towards a younger public, and sometimes non-smokers (Pasquereau et al., 2023). In fact, fruity and sweet flavours are attractive to teenagers and may promote vaping behaviours, thereby increasing the risk of nicotine addiction. This dynamic can be explained by the diversity of flavours offered to consumers, some of which are described as recreational and more appealing than tobacco. E-cig typically comprise three main components: a battery, a heating coil and an e-liquid tank. Several generations of e-cigarettes have been released since their initial launch, but it is mainly the third generation that offers users a personalized experience. In fact, it allows modulation of several key parameters of the e-cig, such as power, voltage and coil heating temperature. In addition to these various parameters, e-liquids are also an important part of the consumer’s experiences. E-liquids are made up of a vehicle classically composed of propylene glycol (PG) and vegetable glycerin (VG), nicotine and flavourings. A significant proportion of consumers use nicotine-free e-liquids, particularly among young people. These users enjoy the recreational aspect of vaping without necessarily being addicted to nicotine, making it essential to investigate the potential toxicity of e-liquid components other than nicotine (Tokle et al., 2022; Jackson et al., 2025). A number of *in vitro* studies have demonstrated that the vehicles and flavours used in e-liquids are involved in toxicity processes (Lerner et al., 2015; Nair et al., 2020; Morris et al., 2021; Effah et al., 2023; Sabo et al., 2023). Indeed, even without nicotine, flavoured e-liquids seem to represent a real risk for several major biological systems, with the pulmonary system in the front line. The extensive range of flavours complicates efforts to assess the toxicity of e-liquids (Krüsemann et al., 2018). Certain aromatic compounds induce dysregulation of the oxidative and inflammatory balance, as well as functional and structural alterations in airway tissues (Lerner et al., 2015; Muthumalage et al., 2018; Nair et al., 2020; Morris et al., 2021; Effah et al., 2023; Sabo et al., 2023; Hwang et al., 2016). In addition to aromatic compounds, there are aerosolization products derived from the oxidation of PG and VG, some of which are known to be toxic or carcinogenic (Kulhánek and Baptistová, 2020; Lai and Qiu, 2020). These *in vitro* investigations have been backed up by several *in vivo* studies highlighting the harmful effects of flavoured and unflavoured nicotine-free e-liquids (Chen et al., 2018; Larcombe et al., 2017; Glynos et al., 2018; Cirillo et al., 2019; Madison et al., 2019; Vivarelli et al., 2019; Kim et al., 2022; 2023; Yanina et al., 2023). Analytical work using gas chromatography coupled with mass spectrometry has revealed the presence of nearly 250 chemical compounds in e-cigarette aerosols (Golpe and Rodríguez, 2023). Some compounds originate from the chemical composition of the e-liquids themselves, while others are degradation products of the vehicle or flavourings (Herrington and Myers, 2015). All these elements can induce harmful cellular responses, such as degradation of the vehicle (PG and VG) or flavourings. The most frequently preferred flavours among e-cigarette users are menthol, followed by tobacco and other fruity flavours (Sargent et al., 2021). It seems essential to identify the different toxicities of fruity e-liquids, which are not well-investigated (Effah et al., 2023; Clapp et al., 2019; Raduka et al., 2023). Here, our aim is to assess the *in vitro* toxicity of a highly popular group of flavours: berry flavours (Strawberry, Raspberry, Blackberry, Blueberry) on an *in vitro* model of the alveolar-capillary barrier (ACB). The latter has been validated phenotypically and functionally. Two methods of exposure to e-liquids (dilution and trapped aerosols) have also been developed and compared to investigate the cellular toxicities. To this end, cell death, barrier integrity and mitochondrial oxidative stress were investigated and described in this work. Finally, an analytical method has been developed using gas chromatography coupled with thermo-desorption and mass spectrometry to identify the compounds present in each e-liquid and to discriminate potentially harmful compounds.

## 2 Materials and methods

### 2.1 Chemical and reagents

#### 2.1.1 Culture cell and tests

RPMI 1640 (#11530586), DMEM (#11574486), SVF (#11573397), Pen-Strep antibiotic (#15140122), GlutaMAX™ (#35050-038), Trypsin (#25200-036), DPBS (#14190144) and MitoSOX® (#M36008) were purchased from Thermo Fisher Scientific (Waltham, United States). Hydrocortisone (#H0888) was obtained from Sigma (Saint-Louis, United States). Lactate Dehydrogenase (LDH) kit (#ab65393), MTS kit (#ab197010) and SP-D SingleStep ELISA® kit (#ab239431) were acquired at Abcam (Cambridge, United Kingdom). Inserts (#83.3932040; #83.3932041) were purchased from Sarstedt (Nümbrecht, Germany).

#### 2.1.2 RT-qPCR

TRIzol™ solution (#15596018) was obtained from Thermo Fisher Scientific (Waltham, United States). Reagents of RT-qPCR were purchased from Bio-Rad (Hercule, United States): iScript™ Reverse Transcription Supermix (#1708841), SsoAdvanced™ Universal SYBR® Green Supermix (#1725271) and primers (Supplementary Table S1).

#### 2.1.3 Western blot

The Bovine Serum Albumine (BSA) (#A9647) and the solution of Triton™ X100 (#T8787) were purchased from Sigma-Aldrich (Saint-Louis, United States). Normal goat serum (#S2000-100) was obtained from Dutscher (Bernolsheim, France). Western blot membranes (#1704157), Precision Plus Protein™ Unstained Protein Standards (#1610363) and Precision Protein™ StrepTactin-HRP Conjugate (#161-0,380) were acquired at Bio-rad (Hercule, United States). Tris Glycine-SDS Buffer 10X (#EU0510) was obtained from Euromedex (Souffelweyersheim, France). Luminata™ solution (#WBLUC0500), Immobilon Classico Western HRP substrate, was purchased from Merck Millipore (Molsheim, France). Antibodies were obtained from Abcam (Cambridge, United Kingdom) or Invitrogen–Thermo Fisher Scientific (Waltham, United States) and are detailed in Supplementary data (Supplementary Table S1).

#### 2.1.4 Immunofluorescence

Formalin solution (#5700), PBS tablets (#18912-014) and some antibodies (Supplementary Table S1) were purchased from Thermo Scientific Fisher (Waltham, United States). Aqueous Mounting Medium with DAPI (#ab104139) and some antibodies (Supplementary Table S1) were obtained from Abcam (Cambridge, United Kingdom).

#### 2.1.5 E-cigarette devices and e-liquids tested

Devices and e-cig liquids were obtained from GAIATREND company (Rohrbach-lès-Bitche, France).

### 2.2 Cell culture

Two different cell lines were used in our study to mimic the ACB. The NCI-H441 cell line (ATCC® - HTB-174™), which is derived from a human papillary adenocarcinoma of the lung, was used as the epithelial cell line. The other cell line used in this study was EA.hy926 (ATCC® - CRL-2922™) established by fusing primary human umbilical vein cells with a thioguanine-resistant clone of A549 by exposure to polyethylene glycol (PEG). EA.hy926 cells were used as lung endothelial cells.

#### 2.2.1 Maintenance of cell lines

NCI-H441 cells were maintained using RPMI 1640 medium supplemented with 10% of fetal bovine serum (FBS) and Pen-Strep antibiotic (0.5%). The cells were sub-cultured twice a week and maintained at a maximum of 25 passages. These epithelial cells were used in the coculture model as type II alveolar cells.

EA.hy926 cells were maintained in DMEM medium supplemented with FBS (10%), Pen-Strep antibiotic (0.5%) and GlutaMAX™ (4 mM). Cells were subcultured up to twice a week and maintained at a maximum of 25 passages. These endothelial cells were used in the coculture model as endothelial cells of the pulmonary microvascularization.

#### 2.2.2 Coculture model

**FIGURE 1:**
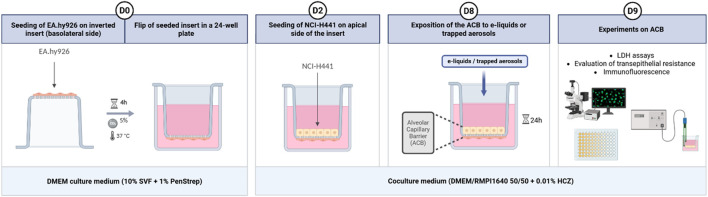
Setting up a coculture model mimicking the alveolar-capillary barrier *in vitro*. Figure shows, chronologically, the various stages involved in setting up the model. At **D0**, EA.hy926 are seeded on an inverted 24-well insert (0.4 µm pore size, PET membrane). Four hours later, the inserts are inverted in a 24-well plate previously filled with culture medium. At **D2**, NCI-H441 cells are seeded in the apical region of the insert in culture medium common to both cell lines. At **D8**, the model can be exposed to e-liquids (diluted or trapped form). Finally, at **D9**, various experiments can be carried out as descript in methods. Created in https://BioRender.com.

A 24-well Transwell insert was used to build the coculture model (Figure 1). The insert consists of a microporous polyethylene terephthalate (PET) membrane with 0.4 µm pore size. The first day of the culture, 30 µL of endothelial cells suspension (EA.hy926 at 1.67 × 10^6^ cells/mL) were seeded on inverted insert. It was left for 4 h at 37 °C and 5% CO_2_ to adhere to the lower part of the insert membrane. The inserts were then inverted and placed in 24-well plates containing 800 µL of DMEM medium in the basolateral region, before 200 µL of DMEM medium were added in the apical region.

Two days later, 200 µL of alveolar epithelial cell suspension (NCI-H441 at 2.5 × 10^5^ cells/mL) were seeded in the apical region of the insert. A coculture medium, containing RMPI 1640 and DMEM medium in a 50/50 ratio and 1 mg/L of hydrocortisone, was deposited in both compartments of the model to facilitate cell growth. The coculture medium was changed every 2 or 3 days until treatments began on day 8.

### 2.3 Characterization of the coculture model

To confirm that our coculture model is indeed a cellular model of the alveolar-capillary barrier, we characterized it phenotypically and functionally.

#### 2.3.1 Phenotypic characterization

Phenotypic characterization was based on the expression and localization of specific proteins directly related to cellular type and function. This is why, we looked for the expression of surfactant proteins (SP-A and SP-D) and junction proteins (Occludin and ZO-1) in epithelial cells and the expression of endothelial NO synthase (eNOS), von Willbrand factor (vWF) and CD31 in the endothelial cells.

##### 2.3.1.1 RT-qPCR

To extract total RNA from the different cell types (EA.hy926 and NCI-H441), a TRIzol solution was used according to the following protocol. After removal of the cell culture medium, the cells were solubilized in TRizol, the total RNAs were then isolated with a chloroform solution, precipitated with isopropanol and washed with a 75% ethanol solution. The amount of RNA extracted was quantified using a NanoDrop™ One (Thermofisher Scientific). Both steps, reverse transcription (RT) followed by amplification (qPCR), were made possible by a Bio-rad thermal cycler (CFX96 - C1000 Touch™). Retro-transcription of total mRNA into cDNA was performed using an iScript™ mix: samples were heated for 5 min at 25 °C, then at 46 °C for 20 min and finally at 95 °C for 1 minute. The final step, amplification of the target sequence using specific primers, was carried out using a SsoAdvanced™ mix containing dNTPs, DNA polymerase and a fluorescent indicator SYBR® Green. The amplification cycle (repeated 45 times) comprises two main stages: cDNA denaturation (30 s at 95 °C), followed by primer hybridization and elongation (30 s at 60 °C). Three reference genes were used to calculate the relative expression of target genes: 18S, GAPDH and beta-Actin. All primers used were obtained from Bio-Rad (detailed in Supplementary Table S1). SP-A, SP-D, ZO-1, Occludin, vWF and eNOS genes were targeted by RT-qPCR.

##### 2.3.1.2 Western blot experiments

Protein expression was studied using the Western blot technique as follows. Cells were lysed with a lysis solution composed of HEPES, EDTA, NaCl and Triton X-100, protease and phosphatase inhibitors. The lysate was centrifuged at 10,000 g for 10 min. The proteins contained in the supernatant were measured using the Bradford method. Proteins were denatured with β-mercaptoethanol and samples containing 20 µg of protein were separated by electrophoresis on an SDS-PAGE gel (10%). Proteins were then transferred on Polyvinylidene fluoride (PVDF) membranes by a Trans-Blot Turbo System (Bio-rad). To block non-specific sites, the membranes were incubated with a solution of Tris-buffered saline solution with Tween 20 and skimmed milk (10%) for 1 hour and then incubated overnight at 4 °C, with the primary antibody, diluted as recommended by the supplier (Supplementary Table S1). The following day, after several washes, the membranes were incubated for 1 h at room temperature with the HRP-coupled secondary antibody, diluted as recommended by the supplier (Supplementary Table S1). The chemi-DocXRS system (Bio-rad) was used to reveal the bands of interest by chemiluminescence after incubating the membranes with Luminata™ as HRP substrate. Using Image Lab software (Bio-rad), the intensity of the bands of interest was analyzed and quantified in relation to the total proteins present in the sample measured by stain-free method, which provides more precise normalization than the methods based on a single housekeeping protein.

##### 2.3.1.3 Immunofluorescence experiments

Cells were fixed with a 4% formaldehyde solution for 15 min. After several washes in PBS, permeabilization and blocking of non-specific sites were performed for 60 min using blocking buffer (5% normal goat serum and 0.3% Triton in PBS). Fixed cells were then incubated overnight at 4 °C with the primary antibody specific to the protein of interest, diluted according to the supplier’s recommendations (Supplementary Table S1) in a PBS solution containing 1% BSA and 0.3% Triton. The following day, after several washes with PBS, cells were incubated with the secondary antibody reactive against the primary antibody species for 2 h and diluted as recommended by the supplier (Supplementary Table S1) in a PBS solution containing 1% BSA and 0.3% Triton. After three final washings, the coverslips were mounted on slides using mounting medium containing DAPI, to label the DNA of cell nuclei. Slides were analyzed using a Zeiss LSM800 confocal microscope. The acquisition conditions (excitation intensity and amplification of the emitted signal) were the same for all samples labelled with the same antibodies.

##### 2.3.1.4 Determination of SP-D protein by ELISA technique

SP-D concentration in NCI-H441 cells was measured by an ELISA (Enzyme-Linked Immunosorbent Assay) kit. This experiment was performed on cell culture supernatant. The coculture was seeded on insert as previously explained. On day 9, the supernatant from the apical region of the insert was collected and stored at −20 °C, to measure the amount of SP-D protein secreted by NCI-H441 epithelial cells. The SP-D protein is captured by an antibody attached to the 96-well plate support, and is recognized by a detection antibody linked to the HRP (Horseradish Peroxidase) enzyme. On addition of the HRP substrate, TMB (3,3′,5,5′-tetramethylbenzidine), an enzymatic reaction generates a signal proportional to the amount of SP-D protein, measurable at 450 nm by spectrophotometry. Concentrations are based on results obtained with the standard range. For each experiment, samples, controls and range points were analyzed in duplicate.

#### 2.3.2 Functional characterization

Functional characterization of the model was based on an assessment of the barrier’s integrity. Trans-epithelial resistance (TER) was chosen as the reference method because it is non-invasive and allows validation of the coculture before further use. TER was also measured after each experiment to measure the impact of the experiment on the ACB model integrity. A decrease in TER values corresponds to a decrease in the barrier’s integrity.

TER was measured with the Evom2® epithelial ohmmeter (World Precision Instruments, Hitchin, United Kingdom) which was connected to standard STX2 electrodes.

### 2.4 Exposure methods

**FIGURE 2:**
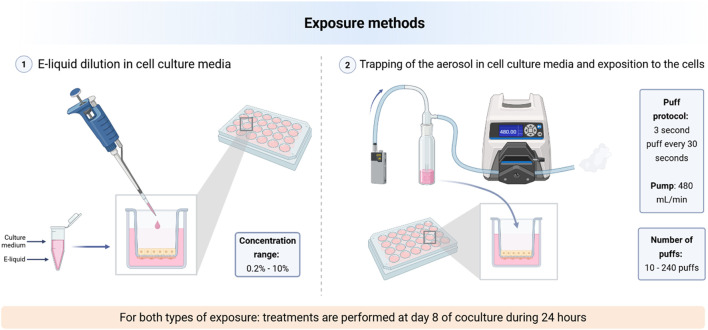
Schematic representation of the two exposure methods used. **(1)** The first involves diluting the e-liquid of interest directly in the culture medium which will be deposited in the apical region. Concentration values vary between 0.2% and 10%. **(2)** The second involves trapping an aerosol in the culture medium (3 s puff every 30 s), using a gas washing bottle connected to an e-cig device and a peristaltic pump, respectively. Then the medium is set as culture medium in apical region. The protocol varies the total number of puffs from 10 to 240 puffs. Created in https://BioRender.com.

To define several toxicities of e-liquids on our cell model of ACB, we compared two exposure methods. The first one was based on the dilution of the e-liquid directly in the cell culture media, whereas the second one consisted of exposing the cells to the aerosol of e-liquid trapped in cell culture media (Figure 2).

#### 2.4.1 Exposure method by e-liquid dilution

This exposure method was based on the dilution of the e-liquid directly in cell culture media: RPMI 1640/DMEM 50/50 for coculture and RPMI-1640 for NCI-H441 monoculture. We tested several concentrations ranging from 0.2% to 10%. After treatment and without medium change, the cells returned in incubator for 24 h, then the end-points experiments (TER, LDH assay) were carried out. Regarding the study of mitochondrial oxidative stress, exposure to diluted e-liquids corresponds to the start of a 1-h kinetic process without prior 24-h incubation. The doses were chosen to cover a wide range of concentrations in order to evaluate potential cellular responses.

#### 2.4.2 Exposure method by trapping the e-cigarette aerosol

This method consisted of trapping the e-cig aerosol in culture medium: RPMI 1640/DMEM 50/50 for coculture and RPMI-1640 for NCI-H441 monoculture. To produce the aerosol, an iStick TC40W electronic cigarette of the Eleaf brand was chosen as well as a RIOK clearomizer containing a mesh coil of 0.7 Ω. The aerosol produced was entrained through tubing into a gas washer bottle by a peristaltic pump Flexicon PF6-B with a flow rate of 480 mL/min. The gas washer bottle was composed of a porosity 2 sintered glass. The aerosolization protocol consists of producing a puff of 3 s every 30 s, varying the total number of puffs from 10 to 240 puffs. After treatment and without medium change, the cells returned in incubator for 24 h, then the end-points experiments (TER, LDH assay) were carried out. Regarding the study of mitochondrial oxidative stress, exposure to trapped aerosol corresponds to the start of a 1-h kinetic process without prior 24-h incubation. The doses correspond to typical consumption among vapers, averaging up to 250–300 puffs.

### 2.5 Tests to evaluate approximate physicochemical alignment between the two methods of exposure

To compare these two methods, we established an approximate physicochemical alignment between the percentage of e-liquid dilution and the number of trapped puffs in medium with different indicators. Thus, for each method, the mass of e-liquid consumed during the protocol was measured. The clearomizer was weighed before and after the aerosolized protocol to evaluate the mass of e-liquid consumed after each puff protocol. A second indicator was the measurement of culture medium osmolality as a function of dilution or number of puffs. This was carried out using the Fiske® Micro Osmometer model 210 (Advanced instruments, Norwood, Massachusetts, United States).

### 2.6 E-liquids of interest

This study used 4 different flavoured nicotine-free e-liquids, all composed of a 76/24 ratio of PG and VG. Four flavours made up these different e-liquids: Strawberry, Raspberry, Blackberry and Blueberry. The e-liquids were obtained from the Alfaliquid® brand (GAIATREND, Rohrbach-lès-Bitche, France).

### 2.7 Cell tests after exposure to diluted e-liquid or trapped aerosol in culture medium

#### 2.7.1 Colorimetric LDH release assay as cytotoxicity indicator

Cytotoxicity was investigated using a LDH assay. This colorimetric method is based on the oxidation by lactate dehydrogenase (LDH) of lactate in pyruvate with the generation of NADH which will itself react with the Water-Soluble Tetrazolium salt (WST) by forming a yellow component. The intensity of the yellow dye is proportional to the number of lysed and therefore non-viable cells. Ten microliters of medium were taken from each well in the apical region of the insert and deposited on a 96-well plate. Next, 100 µL of WST substrate diluted in buffer were deposited per well and incubated with the cell supernatant for 30 min at room temperature. The absorbance was measured at 450 nm using the iMark™ microplate reader (Bio-rad).

#### 2.7.2 MitoSOX® assay as an indicator of mitochondrial oxidative stress

The production of mitochondrial superoxide anions in alveolar epithelial cells was used to investigate the impact of e-liquids in diluted or trapped form at the cellular level. The NCI-H441 cells were seeded at 1 × 10^6^ cells/mL on a 96-well plate with a black background and then placed at 37 °C, 5% CO_2_ for 2 days. The cell medium was removed, and the cells were washed twice with Hank’s Balanced Salt Solution (HBSS). Subsequently, cells were incubated for 30 min with a 5 µM solution of MitoSOX® diluted in HBSS. After two washes with HBSS, the fluorescence reading at 510/580 nm was then programmed on the Varioskan® LUX plate reader (ThermoFisher Scientific): the fluorescence was read every minute to establish the kinetics of the mitochondrial production of superoxide anion. The Area Under the Curve (AUC) was calculated from the kinetic curve and represented as the percentage of the fluorescence intensity measurement over 1 h.

### 2.8 Non-exhaustive qualitative analysis by thermo-desorption coupled with gas chromatography and mass spectrometry (TD-GC-MS)

A volume of 3 μL of each pure e-liquid was collected on a Tenax® cartridge (#N9307005; PerkinElmer) containing a porous polymer matrix. Analysis was conducted using a PerkinElmer ATD-GC-MS 3 device. First, the thermo-desorption step consisted of extracting various volatile compounds from Tenax® cartridge by heating it to 250 °C under a flow of helium for 3 min. The compounds were then trapped in a cooled space at around −30 °C for 1 min to concentrate the analytes before injection. A second step was the vaporization, carried out with increasing temperatures, ranging from 30 °C to 250 °C (at a rate of 40 °C every second) for less than 1 minute to inject the gases generated into the gas chromatograph. The GC part consists of an Elite-WAX column (polyethylene glycol, 30 m, ID 0.25 mm; thickness of 0.25 μm) placed in an oven whose temperature increases gradually to optimize the separation of compounds. This separation is based on the volatility of the chemical compounds and their affinity with the stationary phase (Elite-WAX column). The retention times for each of the compounds analyzed are specific and will enable, in part, their identification. Finally, once separated, the analytes are detected by a mass spectrometer in scan mode, which provides the mass spectrum of each compound. Once the chromatograms and mass spectra have been analyzed, the chemical compounds are identified using a NIST library and only compounds with a high match (>700/1,000) were selected.

### 2.9 Statistical and chemical data analysis

Statistical analyses were performed using GraphPadPrism Version 10 software (San Diego, United States). Statistical analyses of results relating to TER measurements, LDH tests, MitoSOX® tests were performed by a one-factor ANOVA test followed by a Dunett’s multiple comparison post-test and a *p*-value of less than 0.05 was required to consider statistical significance. The lists of compounds found by TD-GC-MS were harmonized in order to eliminate duplicates, standardize chemical names, and ensure comparability between flavours. All analyses were performed using R (version 4.4.2). Data processing was performed using the dplyr package, and proportional Venn diagrams were generated using the eulerr package. The layout of the figures was finalized using the gridExtra package.

## 3 Results

### 3.1 Phenotypical characterization of the alveolo-capillary barrier cell model

The first step of our study was to validate the characteristics of our cell model as an alveolo-capillary barrier model. Specific markers of alveolar epithelium and endothelium were investigated on the 2 cell types by RT-qPCR, Western blot and immunofluorescence.

**FIGURE 3:**
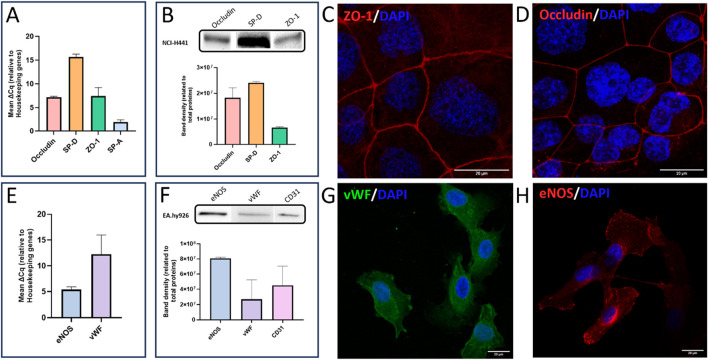
NCI-H441 **(A–D)** and EA.hy926 **(E–H)** cell lines characterization using expression of specific proteins. **(A)** Shows the ΔCq of various mRNA targets used to characterize NCI-H441 (Occludin, ZO-1, SP-A and SP-D) compared with housekeeping genes. **(B)** Shows Western blotting band density on NCI-H441 cells, of three proteins of interest: Occludin, SP-D and ZO-1. **(C,D)** shows NCI-H441 cells in which respectively ZO-1 and Occludin are labelled with Alexa-647® (red). **(E)** Shows the ΔCq of the mRNA targets used to characterize EA.hy926 (vWF and eNOS) compared with housekeeping genes. **(F)** Shows Western blotting band density of three proteins of interest: eNOS, vWF and CD31. **(G,H)** show EA.hy926 cells in which vWF, an endothelial protein, is labelled with Alexa488® (green) and eNOS, an enzyme expressed by the vascular endothelium, is labelled with Alexa647® (red), respectively. All images were captured with a confocal microscope, nuclei were labelled with DAPI (blue) and scale bars are 20 µm. Histograms results are mean from 3 separate experiments of triplicates ±SEM. Western blot histogram normalizes the signal with stain-free technology (total proteins).

As shown in Figure 3, ZO-1 and Occludin, two tight junction proteins, were expressed and identified in NCI-H441 cells (Figures 3A–D). ZO-1 was specifically and only present on the membrane of the cells as expected (Figure 3C). The same pattern was observed for Occludin (Figure 3D). SP-A, surfactant protein A, was expressed in NCI-H441 cells on mRNA level and SP-D, surfactant protein D, was expressed on mRNA and protein level (Figures 3A,B). The concentration of SP-D protein was measured by ELISA in NCI-H441 monoculture. The average was around 53 pg/mL. It should be noted that the concentration of SP-D measured in the coculture averaged 172 pg/mL and was significantly higher than in monolayer of NCI-H441 cells (Supplementary Figure S2– Supplementary data).

The expression and localization of the von Willebrand factor (vWF), a coagulation factor, were observed only in EA.hy926 cells. The vWF protein was localized in cytoplasm as expected and shown in Figure 3G. As eNOS, principally expressed in the vascular endothelium, is one of the three isoforms that synthesize nitric oxide (NO), its presence was successfully identified in endothelial cells as shown in Figures 3E,F,H. Finally, CD31, the last endothelial marker tested, was identified in EA.hy926 cells as shown in Figure 3F.

A 3D reconstruction of the coculture using confocal microscopy allowed us to confirm the presence of junction proteins (occludin) in the membrane region of NCI-H441 cells and proteins specific to endothelial cells in the cytoplasmic region of EA.hy926 cells on day 8 of coculture (Supplementary Figure S3).

### 3.2 Functional characterization of the alveolo-capillary barrier cell model

**FIGURE 4:**
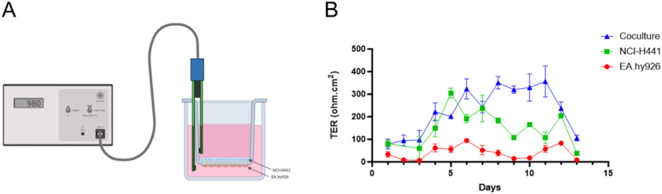
Functional assessment of the barrier *in vitro* by measuring transepithelial resistance (TER) values (Ohm.cm^2^) over time (days) under three different conditions. **(A)** Represents the method used to measure TER. **(B)** Represents TER values obtained for coculture (blue), NCI-H441 cells alone (green) and EA. hy926 cells alone (red) over time. Values represent mean ± SEM from 3 separate experiments of triplicates. Created in https://BioRender.com.

The second step of our study was to validate the function of our cell model as an alveolar-capillary barrier model. This was tested by measuring TER values every day in mono and coculture conditions (Figure 4A). As shown in Figure 4B, coculture was most effective as a barrier and over a longer time (6 days), followed by epithelial cells whose effectiveness was shorter in time (only 3 days). The stable TER values were between 300 and 400 Ω.cm^2^ from D6 and D11 for the coculture. Epithelial cells play an essential role in barrier function of the coculture. Based on these results, all experiments were carried out at D8 and only if the TER of the coculture was greater than 300 Ω.cm^2^.

### 3.3 Approximate physicochemical alignment between the two methods of exposure

**FIGURE 5:**
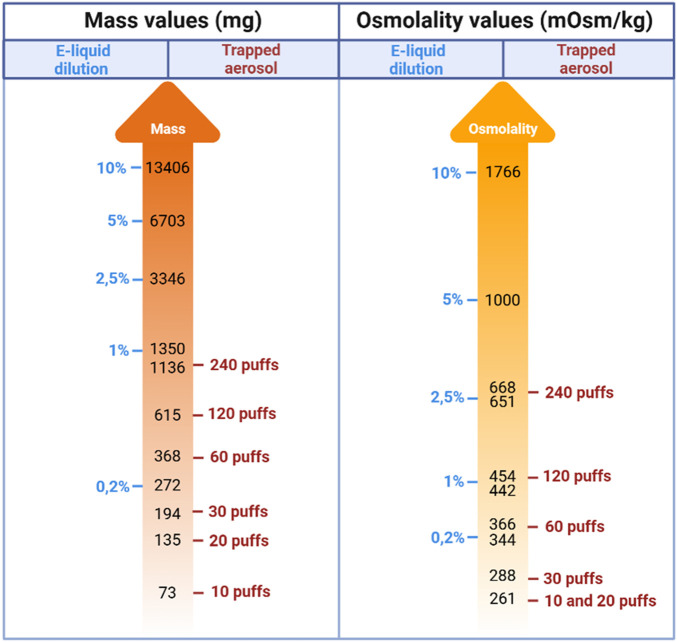
Schematic representation of the approximate physicochemical alignment calculated between the dilution and aerosol entrapment exposure methods, based on two different parameters: mass (mg) and osmolality (mOsm/kg). The results are presented as a logarithmic scale (Log_10_) and represent the mean of the results obtained for each flavoured e-liquid (Strawberry, Raspberry, Blackberry, Blueberry). Created in https://BioRender.com.

For the method e-liquid dilution model in the cell culture medium, the molecular concentrations were known for all the components present in the e-liquid, which was not the case for the method of trapped aerosol of the e-liquid. For this reason, we measured two markers in the cell culture medium for each type of exposure. The results presented in Figure 5 show that 240 trapped puffs approximatively represent the equivalence of 1% of e-liquids vehicles mass, but correspond with over 2.5% of vehicle osmolality. Overall, these results show that a large number of puffs were required to achieve approximate physicochemical alignment for a low concentration of diluted products.

### 3.4 Impact of exposure on the cell viability

LDH is a cytoplasmic enzyme expressed constitutively in all cell types and it is released into the culture medium during cytolysis. Measurement of absorbance (450 nm) of the LDH products after exposure to increased e-liquid concentrations by dilution (from 0.2% to 10%) or trapping of aerosol (from 10 to 240 puffs) was not modified compared to untreated cells (control), except for Blueberry flavoured e-liquid (Supplementary Figure S4). In fact, for low concentrations of Blueberry flavoured e-liquids diluted in culture medium (0.2%–2.5%), a significant decrease in LDH release and probable cell death was observed. A significant increase in LDH release was observed with the PG/VG vehicle (*p* < 0.05), along with a tendency for diluted flavoured e-liquids to induce higher LDH levels at a concentration of 10%.

### 3.5 Impact of exposure on the barrier permeability

**FIGURE 6:**
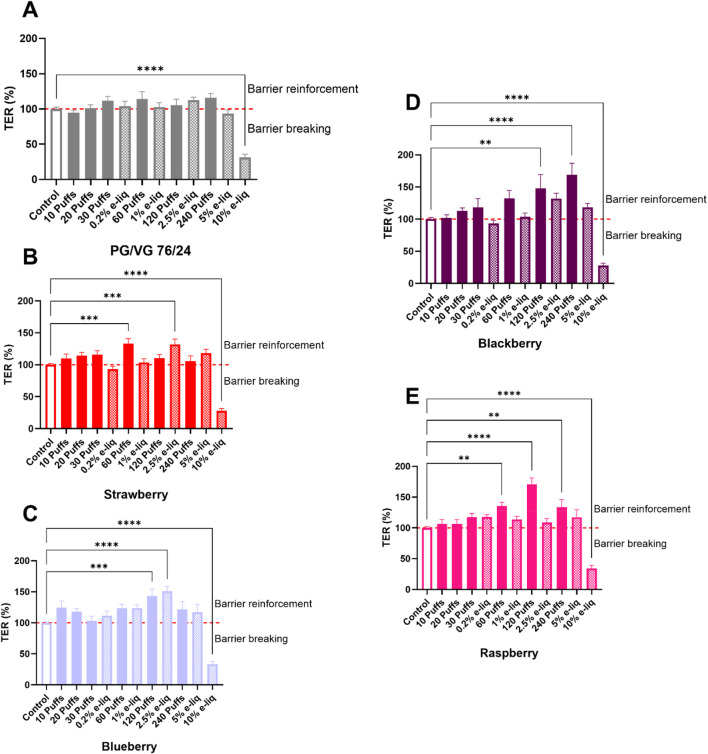
Measurement of transepithelial resistance (TER) in percentage of control (controls are white histograms and arbitrary set at 100%) after a 24 h exposure to e-liquids by dilution (dashed histograms) or trapped (full colored histograms). The exposures are classified according to their osmolality values. Each graph represents a specific flavoured e-liquid. **(A)** Represents TER in percentage of control after an exposure to PG/VG (76/24) exposure in culture medium by dilution (%) or with trapped aerosol (puffs). **(B–E)** Represent TER in percentage of control after an exposure to PG/VG (76/24) + Strawberry, Blueberry, Blackberry and Raspberry flavours by dilution (%) or with trapped aerosol (puffs), respectively. Data are shown as mean ± SEM, *n* = 3–4 experiments of triplicates. Statistics: One-way ANOVA, followed by Dunnett’s multiple comparisons test; α = 0.05 (**: *p* < 0.01; ***: *p* < 0.001; ****: *p* < 0.0001).

Increasing the osmolality of the culture medium is a factor in cell retraction, which can lead to permeabilization or rupture of the ACB model. The molecules influencing osmolality are mainly the PG and VG of the vehicle. The ACB significantly increased its permeability for a dilution of PG/VG higher than 5% of liquid or higher than 1,000 mOsm/kg (Figures 5, 6). It is interesting to note that the dilution of small quantities of e-liquid (0.2%–2.5%) in the cell culture medium has no impact on the permeability of the barrier except for a 2.5% dilution of e-liquid containing Strawberry or Blueberry flavours. In the latter two cases, the TER values were significantly increased meaning that the barrier was likely strengthened.

In our experiments with trapped aerosols, the TER value did not decrease due to the low trapping of PG/VG in the culture medium after the generation of 10–240 puffs. More surprisingly, in cell medium containing trapped aerosol, the berries TER profile was different compared to PG/VG profile, insofar as an increase in TER was observed with all the berries but with different intensities and depending on the number of puffs. Thus, for the Strawberry and Raspberry aerosols emerged a significant increase of TER from 60 puffs, whereas the other berry flavours only induced a significantly increase of TER from 120 puffs. Moreover, Blackberry and Raspberry trapped aerosols presented a maximum of values up to 169% and 171% of the control, respectively.

### 3.6 Impact of exposure on mitochondrial oxidative stress

**FIGURE 7:**
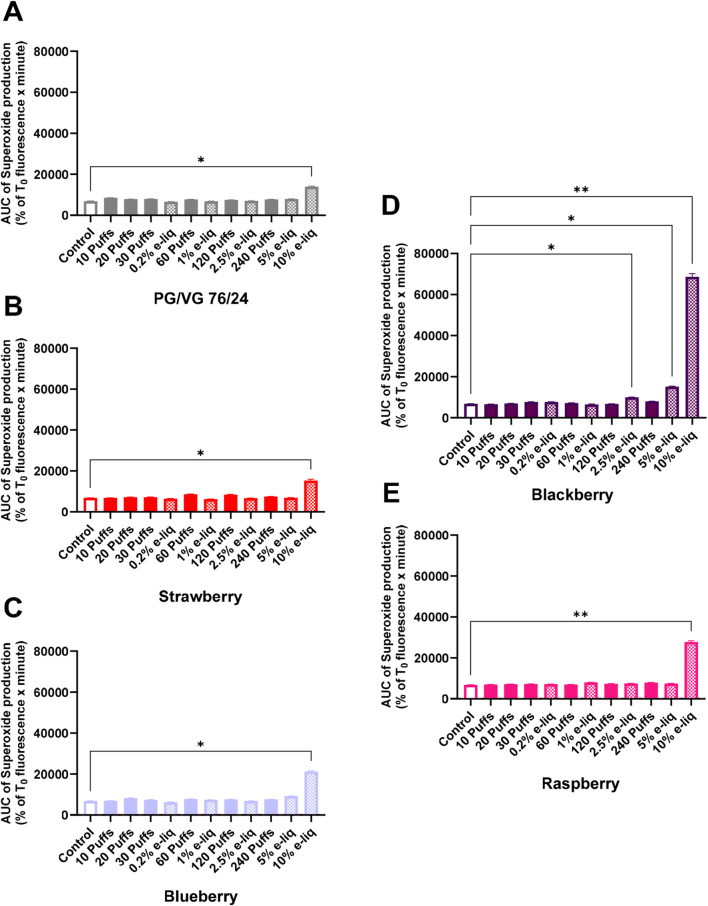
Area under the curve (AUC) of superoxide production according to exposure of e-liquid by dilution (dashed histograms) or by trapped aerosol (full colored histograms) containing **(A)** PG/VG alone without flavours, **(B)** PG/VG (76/24) + Strawberry flavours **(C)**, PG/VG (76/24) + Blueberry flavours **(D)** PG/VG (76/24) + Blackberry flavours **(E)** and PG/VG (76/24) + Raspberry flavours. These AUC represent values of fluorescence curve kinetic, for 1 h, obtained with a Mitochondrial Superoxide Indicator (MitoSOX Red®). Data are shown as mean ± SEM, *n* = 3–4 experiments of triplicates. Statistics: One-way ANOVA, followed by Dunnett’s multiple comparisons test; α = 0.05 (*: *p* < 0.05; **: *p* < 0.01).

Berry flavours are often associated with antioxidant properties; therefore mitochondrial superoxide anion production was measured for each exposure to the different flavours (Figure 7). The results shown in Figure 7 indicated that PG/VG do not generate significantly more superoxide anion than the control (untreated cells) except at a high concentration in the diluted form. In fact, the production of anion superoxide was significantly higher than the control at 10% of e-liquid (*p* = 0.0418). Strawberry (*p =* 0.0167), Raspberry (*p =* 0.0069) and Blueberry (*p =* 0.0106) flavours showed the same oxidative stress profile than the vehicle. However, we were able to observe an increase in superoxide production for lower concentration for the Blackberry flavour compared to the vehicle and the other e-liquids. Indeed, mitochondrial oxidative stress increased in a concentration-dependent manner in the diluted form, reaching significance at 2.5% (*p =* 0.0459) and further increasing at 5% (*p =* 0.0171) and 10% (*p =* 0.0021). However, whether for the vehicle alone or for flavoured e-liquids, no significant increase was found for the trapped form.

### 3.7 Comparison of the chemical signatures of e-liquids: common compounds and compounds specific to flavours

**FIGURE 8:**
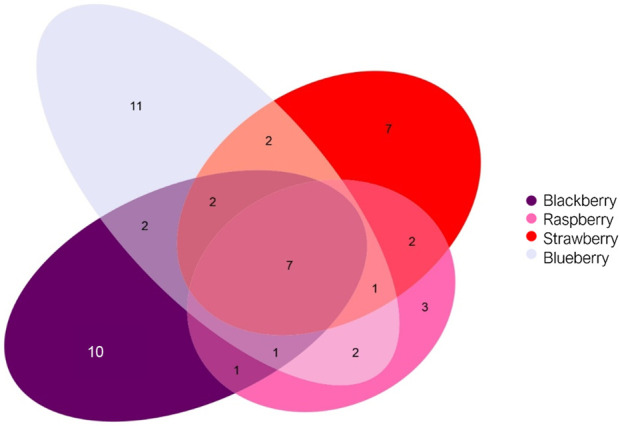
Venn diagram representing the chemical compounds identified in the four flavoured e-liquids (Blackberry, Raspberry, Strawberry, and Blueberry). The non-overlapping areas indicate compounds specific to each flavour, while the overlapping areas represent compounds shared between several flavours. The size of the ellipses is proportional to the total number of compounds detected in each e-liquid. The values shown correspond to the number of compounds present in each category.

The responses observed during the various toxicity tests suggest differences in toxicity depending on the chemical composition of the flavourings in question. The chemical signatures of the various berry-flavoured e-liquids were analyzed by TD-GC-MS. After analyzing the chromatograms, we identified seven compounds common to all four e-liquids: acetaldehyde, ethanol, isopropyl acetate, hexyl alcohol, cis-3-hexenol, propylene glycol, and glycerin. In addition to these compounds, various elements specific to each flavour were identified, listed in Table 1. These results are illustrated in Figure 8 in the form of a Venn diagram. Based on these results, the Blackberry and Blueberry e-liquids have more compounds specific to them (10 and 11 compounds respectively), while the Strawberry and Raspberry e-liquids have fewer specific compounds (7 and 3 compounds respectively). It seems that the more compounds e-liquids contained, the more they caused cellular responses like mitochondrial stress, barrier disruption or cell death.

**TABLE 1:** Comparative aromatic profile: common chemical compounds and compounds specific to each e-liquid flavour (Blackberry, Raspberry, Strawberry, Blueberry).

Common compounds	Blackberry	Raspberry	Strawberry	Blueberry
Acetaldehyde Ethanol Isoamyl acetate Hexyl alcohol Cis-3-Hexenol Propylene glycol Glycerin	1-(Methoxymethoxy)hexane 1-O-Methylglycerol 2-Nitroethyl propionate 2-Hexen-1-ol 3,3,4,4-Tetrafluorohexane Formic acid Methanesulfonyl chloride Amyl formate Maltol Allyl propanoate	2-Propanol, 1-(2-propenyloxy) 3,3,4,4-Tetrafluorohexane Propanal	2-Methylbutyric acid Benzoic acid Isoamyl isovalerate Ethyl lactate Limonene Pentanal Succindialdehyde	2-Propanol, 1-(1-methylethoxy)-α-Keto-butyric acid (Z)-3-Hexenyl acetate Ethyl glycolate Hydroxyacetone Isoamyl isobutyrate Methyl butyrate Methylglyoxal Pentyl formate Trans-β-Damascone α-Ionone

## 4 Discussion

The aim of this study was to highlight the potential toxicity and effects of four berry flavours on an *in vitro* model of the alveolar-capillary barrier. Two exposure methods were investigated: the first involved diluting the e-liquid in the cell medium, while the second involved exposing the model to an aerosol trapped in culture medium.

Firstly, the coculture model was validated both phenotypically and functionally, as this model had never been described in publication. To this end, several key markers of the cells making up the alveolar-capillary barrier were targeted, such as cell junction proteins, surfactant protein A and D for alveolar epithelial cells, and endothelial specific proteins such as CD31, vWF and eNOS for endothelial barrier cells. We confirmed the expression of these markers by RT-qPCR, Western blot and immunofluorescence. We also determined the amount of SP-D released by the cells in the cell supernatant in the apical region of the coculture insert. We found an approximate concentration of 171 pg/mL of SP-D protein by an ELISA assay. This concentration appears to be much lower than that found in the literature for serum or bronchoalveolar lavage assays on healthy patients. In serum, concentrations between 66 and 107.7 ng/mL have been described (Honda et al., 1995; Grosicka et al., 2018). In bronchoalveolar fluids, SP-D concentrations are much higher, approaching 800 ng/mL (Honda et al., 1995). However, the literature is fairly thin on ACB cellular models and the concentrations of SP-D that can be released by the cells. One recent study showed that SP-D concentration in airway surface fluids was around 17 ng/mL in a primary model of alveolar epithelial cells after 14 days in Air Liquid Interface (ALI) (Tanabe and Ishikawa, 2025). We assume that the low SP-D concentration in our model can be explained by different ways. Firstly, it is possible that the use of a cell line in the coculture model rather than primary cultures from patients is a factor that may account for the low concentration of SP-D measured in the surfactant. It is possible that NCI-H441 cells, although considered as type II pneumocytes, are not 100% identical to type II alveolar cells from a functional and secretory point of view (Brookes et al., 2021). Secondly, the absence of an immune component in the coculture model limits the influence of cytokine stimuli on NCI-H441 epithelial cells and may limit surfactant protein expression. Surfactant protein expression appears to be dependent on several types of signal in the pulmonary region (Bein et al., 2013; Ito and Mason, 2010). Indeed, one study demonstrated the impact and influence of certain Th1/Th2 cytokine signals on human type II alveolar epithelial cells. IL-13 significantly reduced the expression of mRNA encoding the SP-D protein, while IFN-γ significantly increased mRNA and protein expression (Ito and Mason, 2010). It could therefore be interesting to evaluate the SP-D concentration after treatment with cytokines, or to add PMA-treated THP-1 monocytic cells to the coculture model to confer macrophagic properties (Wang et al., 2020). Finally, the low concentration measured in the supernatant of our cocultures may be linked to a phenomenon of dilution of a low protein concentration in the volume of medium placed in the apical region of the insert. Furthermore, this culture medium is changed every 2 days, whereas type II alveolar epithelial cells are known for their particularly slow secretory functions (Haller et al., 1998). It is therefore possible that the cells did not have time to secrete and accumulate enough SP-D protein in medium before sampling. Although the model was well characterized using these techniques, the use of immortalized cell lines remains a limitation and must be taken into account when interpreting these results.

Once the cellular model had been developed, the two methods for exposing the coculture model to e-liquids/aerosols were set up. To compare the two methods, two different markers were used: the mass of e-liquid aerosolized/diluted and the osmolality of the culture media in which the e-liquids were diluted, or the aerosols were trapped. The osmolarity of the media was used as a marker of approximate physicochemical alignment, as described in several studies. In fact, e-liquids have a hyperosmolar character, mainly due to the PG/VG vehicle (Gonzalez-Suarez et al., 2017; Munakata et al., 2018; Czekala et al., 2019; Sabo et al., 2023). For this reason, we decided to use this marker to compare the two exposure methods. The links between the 2 methods of exposure were not straightforward, as aerosolization does not entrain all the molecules present in the e-liquid in the same way. In addition to the difficulty of comparing these methods in terms of concentration, it is also difficult at present to establish the proportion of elements present in the e-liquid that have been successfully transferred in the culture medium after trapping. Indeed, the various components of an e-liquid, including flavours, have different evaporation characteristics, so it is conceivable that a portion of the compounds may not be trapped in the culture medium. In our study, osmolality is a factor that focuses mainly on the vehicle and its hyperosmolar properties, but it cannot be ruled out that other compounds may also play a role, such as flavouring compounds and thermoformed compounds during aerosolization. Several other factors can also influence the trapping of chemical compounds in the culture medium, including the polarity, composition of the vehicle used in the e-liquid, but also the solubility of compounds in the vehicle (Noël et al., 2019). Overall, these results show that a large number of puffs are required to achieve an approximate physicochemical alignment with a low concentration of diluted products. In fact, to reach a concentration of 2.5% by dilution of e-liquid in culture medium, *via* the physicochemical alignment established by osmolality, we estimate that around 240 puffs must be trapped in the culture medium. Inhaling 240 puffs represents a day’s vaping for a moderate user. It is known that 1 mL of e-liquid corresponds to approximatively 300 puffs (Hildick-Smith et al., 2015). It is recommended that daily consumption should never exceed 5 mL/day or 1,500 puffs/day. In addition to the number of puffs, other factors such as e-cig power, inhalation profiles and the vehicles used can significantly vary the use and doses inhaled by consumers.

We showed in the results that LDH concentration, linked to cell death, was no significantly changed after treatment with e-liquids or trapped aerosols except for Blueberry flavoured e-liquid which caused a decrease in cell death at low concentrations of diluted e-liquid. We have, however, identified a tendency for diluted e-liquids to increase cytotoxicity in high concentration in the culture medium (10%). This observation was also described in the work of Sabo et al. where a significant increase in cell death, by LDH assay, was demonstrated (Sabo et al., 2023). The hyperosmolarity of the cellular environment, due to PG/VG vehicle in a flavour independent way, may be at the origin of an osmotic shock that cannot be compensated by cellular mechanisms. For Blueberry-flavoured e-liquid, the decrease in cell death at low concentrations may be explained by the presence of antioxidative compounds, particularly anthocyanins, which are present in higher amounts in blueberries compared to the other fruity flavour studied (Wu et al., 2006). However, this decrease in LDH should be interpreted with caution, as previous studies focusing on e-liquids have described interference during colorimetric enzyme tests of this type (Chhor et al., 2023).

We can compare these cell death observations with those obtained when assessing barrier integrity. For high concentrations of e-liquids diluted in cell culture medium (10%), we have significant cell responses. Indeed, we have demonstrated a significant reduction in trans-epithelial resistance, TER, and thus in barrier integrity *in vitro*. These results confirm the observations made concerning cell death, and have also been described by Sabo et al. (2023). Decreases in barrier integrity have already been described after exposure to diluted e-liquids, but also to aerosols exposed directly to the cells. This was described by Effah et al. after exposure to aerosols composed of PG/VG vehicles with or without flavourings (hazelnut, cinnamon, *etc.*) (Effah et al., 2023). However, according to the physicochemical alignment scale established in this work, 10% of e-liquids would be equivalent to 1,000 puffs. These daily amounts are rarely inhaled by vapers, as discussed above. However, our results show a significant increase in TER for each of the flavoured e-liquids at intermediate concentrations. Indeed, at 60 puffs, TER increased significantly for both Strawberry and Raspberry flavours. TER increased significantly for Blackberry, Raspberry and Blueberry flavours at 120 puffs, and at 240 puffs for Blackberry and Raspberry. These differences on barrier integrity can be explained by the differences in chemical composition of the flavours, which may lead to different processes in the cellular model. Another hypothesis can also be the different volatility of the compounds and their ability to be transferred to the culture medium for aerosol trapping. In addition to exposure by aerosol trapping, exposure to e-liquids also revealed an increase in TER at 2.5% for Strawberry- and Blueberry-flavoured e-liquids. This observation was also highlighted in the paper by Sabo et al., where e-liquid diluted to 2.5% in the culture medium caused a significant increase in TER for another coculture model of the ACB (Sabo et al., 2023). This increase appears to be dependent on the flavours added to the vehicles, as no significant increase was observed in TER after exposure to the PG/VG vehicle alone (76/24) by aerosol dilution or trapping. However, it cannot be ruled out that other mechanisms may indirectly contribute to the increase in TER, such as cellular morphological changes, alterations in ion transport, or changes in the conductivity of the medium. In order to explain this unexpected increase in TER, it seems worthwhile to study the expression of several junction proteins directly linked to the integrity of the ACB, such as occludin, ZO-1 or claudin-5 and also, morphological change of cells or expression of main ionic transporters.

Finally, we studied mitochondrial oxidative stress in NCI-H441 cells after exposure to diluted e-liquids and trapped aerosols. Once again, differences between flavour types are notable. 1n fact, we observed for the Blackberry flavoured e-liquid an increase in mitochondrial oxidative response for lower concentration compared to the vehicle alone and other flavoured e-liquids. The chemical composition of the aromas may once again explain these differences. We have also shown that, compared with the diluted form, the trapped form does not significantly increase mitochondrial oxidative stress. We can assume that the aerosol trapping method does not necessarily contain all the elements present in the e-liquid, or it does so in lesser proportion, which supports what has been demonstrated in the comparison of the two exposure methods. In addition, direct exposure of cells to e-cigarette aerosol produced in a standardized way by a smoking machine would also be a further step towards limiting exposure bias and mimicking more closely what happens *in vivo* in vapers.

Lastly, we highlighted the impact of the chemical composition of flavoured e-liquids on the cellular responses observed, mainly concerning mitochondrial oxidative stress. To do this, we studied the chemical compositions of e-liquids using a TD-GC-MS method in a non-exhaustive, probabilistic and qualitative manner. The results obtained enabled us to highlight differences in composition depending on the flavour considered. These analyses allowed us to determine specific flavouring compounds in each e-liquid (Blackberry, Blueberry, Strawberry, and Raspberry). In the case of Blackberry e-liquid, for which we found the highest toxicity in terms of mitochondrial oxidative stress, we assume that one or more chemical compounds could be potentially responsible for these harmful responses. Indeed, 2-Nitroethyl propionate and Methanesulfonyl chloride, identified only in the Blackberry e-liquid, are described as toxic compounds when inhaled and could be responsible for the increased toxicity observed in the MitoSOX® test (PubChem, 2005b; PubChem, 2005a). However, in order to confirm these hypotheses, it would be essential to conduct an in-depth study using analytical standards and quantitative analysis to establish the actual amount of these potentially harmful compounds present in e-liquids and to ensure that these compounds are not artefacts resulting from the chromatography analysis method or NIST base research. It would also be interesting to treat the cell model with different identified flavouring compounds in a PG/VG ratio in order to study their specific effects from a toxicological point of view.

## 5 Conclusion

In conclusion, this work led to the design and validation of a new *in vitro* model of the alveolar-capillary barrier, with the aim of studying the toxicity of e-liquids widely used by consumers: berry-flavoured e-liquids. To do this, we used two methods of exposing the coculture model, one by dilution and the other by aerosol trapping, which allows us to get closer to the composition of the e-liquid actually inhaled. We were able to highlight the hyperosmolar nature of the vehicle contained in the vast majority of e-liquids. This hyperosmolar feature seems to be at the origin of various cellular responses such as an increase in cytotoxicity, an alteration in barrier integrity and an increase in mitochondrial oxidative stress at high concentration. We were able to demonstrate different cellular responses depending on the exposure method used. In fact, for the trapped form of the aerosol, we demonstrated an increase in barrier integrity at low number of puffs. In addition to exposure methods, we found variable cellular responses depending on the type of flavouring present in the e-liquid. Blackberry flavour seems to be more oxidant than other berry flavours. To provide further answers to these inter-flavour and inter-exposure method variabilities, we characterized chemically the 4 e-liquids studied. The analyses performed appear to highlight distinct chemical compositions that may be responsible for the varied cellular responses observed. The specific effect of the molecules implicated still needs to be investigated.

## Data Availability

The original contributions presented in the study are included in the article/Supplementary Material, further inquiries can be directed to the corresponding authors.
