# Supplementary material for: Berry e-liquid flavour toxicities are not equal on an alveolar-capillary barrier cell model according to two exposure methods
Source: Front Toxicol. 2026 Mar 11;8:1769275. doi: 10.3389/ftox.2026.1769275 (PMC13012864; doi:10.3389/ftox.2026.1769275)
Supplement: Supplementary file 1 [file DataSheet1.pdf]

Supplementary Data

1 Supplementary Data

1.1 Supplementary Table

Table S.1: List of primers and antibodies

Primer	Reference	Brand
18S	#qHsaCED0037454	Bio-rad
Actin	#qHsaCED0005010	Bio-rad
GAPDH	#qHsaCED0038674	Bio-rad
ZO-1	#qHsaCID0018062	Bio-rad
Occludin	#qHsaCED0038290	Bio-rad
SP-D	#qHsaCID0012776	Bio-rad
SP-A	#qHsaCED0005710	Bio-rad
vWF	#qHsaCED0043330	Bio-rad
eNOS	#qHsaCID0015042	Bio-rad

Primary antibody	Reference	Brand	Dilution
Mouse Anti ZO-1	#33-9100<br>RRID: AB_2533147	Invitrogen	WB: 1000<br>IF: 1/100
Mouse Anti Occludin	#33-1500<br>RRID: AB_2533101	Invitrogen	IF: 1/100
Rabbit Anti Occludin	#ab216327<br>RRID: AB_2737295	Abcam	WB: 1/1000<br>IF: 1/100
Mouse Anti SP-D	#ab17781<br>RRID: AB_444000	Abcam	WB: 1/500<br>IF: 1/100
Rabbit Anti CD31	#ab28364<br>RRID: AB_726362	Abcam	WB: 1/500<br>IF: 1/100
Rabbit Anti vWF	#ab6994<br>RRID: AB_305689	Abcam	WB: 1/500<br>IF: 1/100
Mouse Anti eNOS	#ab76198<br>RRID: AB_1310183	Abcam	WB: 1/1000<br>IF: 1/100

Secondary antibody	Reference	Brand	Dilution
Anti Mouse IgG-HRP (produced in Rabbit)	#A9044<br>RRID: AB_258431	Sigma-Aldrich	1/10000
Anti Rabbit IgG-HRP (produced in Goat)	#A9169<br>RRID: AB_258434	Sigma-Aldrich	1/10000
Anti Rabbit IgG-Alexa Fluor™ Plus 488 (produced in Goat)	#A32731<br>RRID: AB_2633280	Invitrogen	1/500
Anti-Mouse IgG-Alexa Fluor® 647 (produced in Goat)	#ab150115<br>RRID: AB_2687948	Abcam	1/500

1.2 Supplementary Figures

Figure S.2

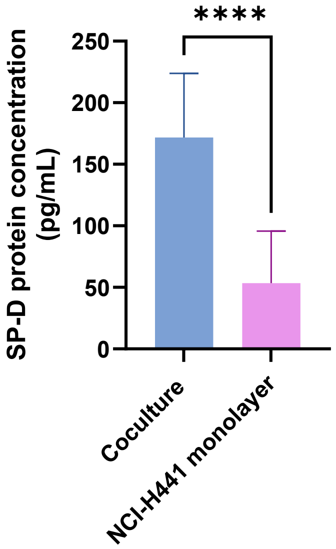

Figure S.2. Histograms representing the SP-D protein concentration in pg/mL determined by an ELISA test in the supernatant of the coculture or monolayer of NCI-H441 cells. Data are shown as mean ± SD, n = 2 experiments of triplicates. Statistics: t-test; $\alpha = 0.05$ (****: $p < 0.0001$)

Figure S.3

(MP4 format video)

Figure S.3. Three-dimensional (3D) reconstruction of the insert-based coculture model at day 8 (D8). The apical cell layer is shown in red (occludin, a tight junction protein), while the endothelial cell layer is shown in green (CD31, a specific marker of endothelial cells). Cell nuclei are stained in blue. The unlabeled space between the two cell layers corresponds to the insert membrane, which is not stained. Images were acquired as Z-stacks using a confocal microscope (Zeiss LSM800), and 3D reconstruction was performed using Imaris software. Scale bar: 10 µm.

Figure S.4

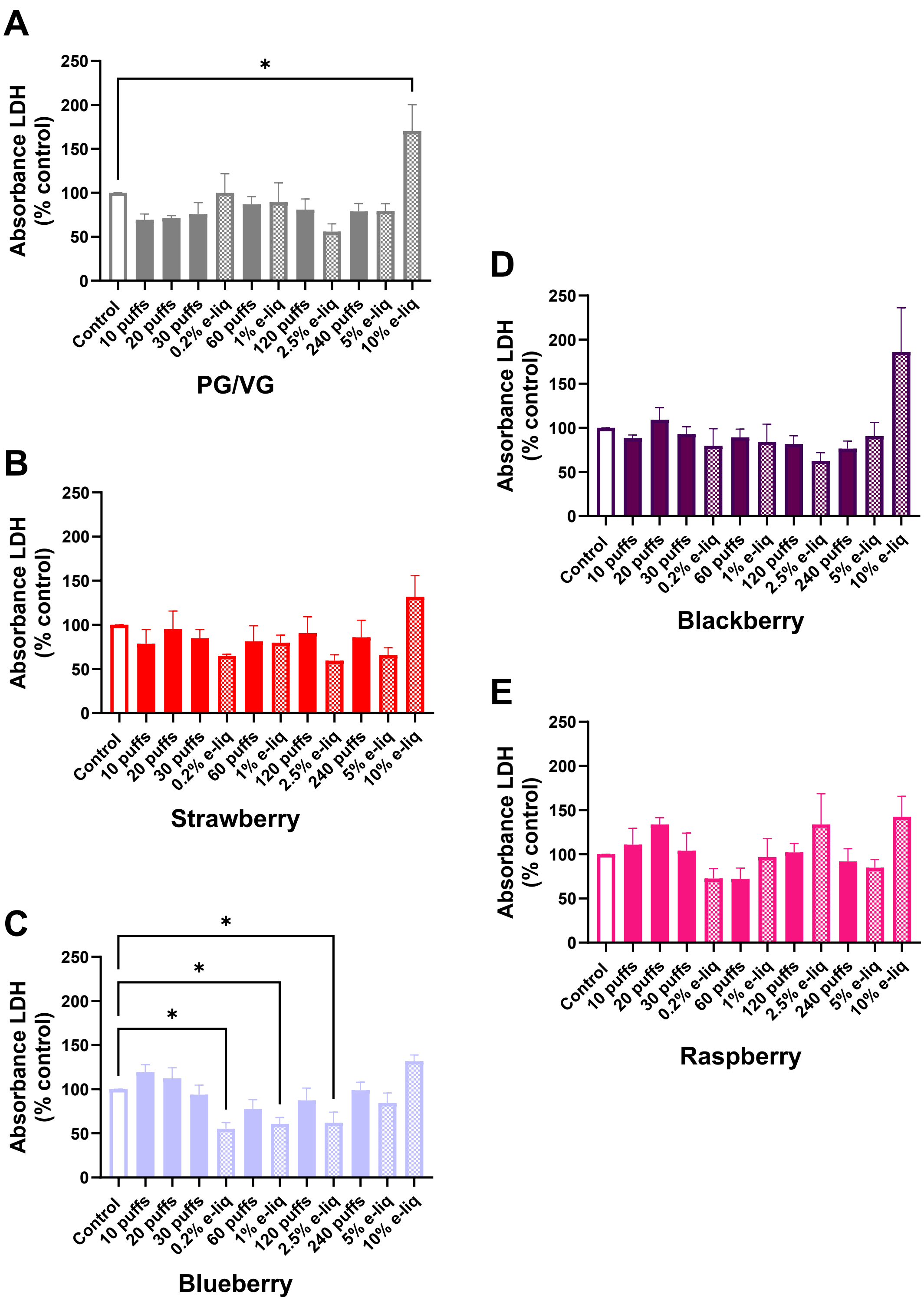

Figure S.4. Measurement of LDH absorbance (450 nm) according to e-liquid concentration. Each graph stands for a specific flavoured e-liquid. (A) represents LDH absorbance in percentage of control

after an exposure to PG/VG (76/24) exposure in culture medium by dilution (%) or with trapped aerosol (puffs). (B) to (E) represent LDH absorbance in percentage of control after an exposure to PG/VG (76/24) + Strawberry, Blueberry, Blackberry and Raspberry flavours by dilution (%) or with trapped aerosol (puffs), respectively. Controls are untreated cell, white histograms and arbitrary set at 100%. Exposure of e-liquid by dilution is represented as dashed histograms and by trapped aerosol as full colored histograms. Data are shown as mean ± SEM, n = 3-4. Statistics: One-way ANOVA, followed by Dunnett's multiple comparisons test; α = 0,05 (*: $p < 0{,}05$)
